# Note on Smoked Glasses

**Published:** 1895-06

**Authors:** Richard H. Satterlee

**Affiliations:** 189 Delaware Avenue; Buffalo, N. Y.


### NOTE ON SMOKED GLASSES.

By RICHARD H. SATTERLEE, M. D., Buffalo, N. Y.

Eyestrain as a potent factor in the causation of headaches and general nervous phenomena is admitted by the most conservative. How necessary then, in these cases, to eliminate this element whenever found. A frequent cause of eyestrain, that is often overlooked, is the cheap smoked glass.

The glass I refer to is an imitation of the French coquilles. When properly made, there is no objection to coquilles, but they are expensive and hard to find. The cheap American imitation has curved surfaces, the convex surface being out and the concave surface worn next to the eye. Usually the concave surface exceeds the convex surface in curvature and the result is a nearsighted lens. This can readily be proved by holding the glass in front of the eye and moving it back and forth. If the object, as seen through the glass, seems to retreat ever so slightly when the lens is moved away from the eye of the observer, the lens is a nearsighted glass. Often it will be found that if the glass is held parallel with the eye and rotated slightly, all objects seen through the glass will be distorted. This shows imperfections in the curvature, which would tend to irritate the eyes whenever worn.

Any bright light causes pain, because it produces extreme contraction of the pupil, and this strain on the ciliary muscle tires the eyes in a short time. A smoked or tinted glass shuts out some of the rays of light and allows the pupil to dilate, producing at once a sense of comfort.

The majority of people wearing these smoked glasses are farsighted. Wearing these curved American nearsighted glasses brings the accommodation into play, creating all sorts of reflex nervous disturbances, and may even permanently impair the vision.

The smoked or tinted glass with plane surfaces are cheap and do no harm whatever to the eyes. This kind should always be preferred.

189 Delaware Avenue.

